# Long non-coding RNA LINC00152 promotes cell proliferation, metastasis, and confers 5-FU resistance in colorectal cancer by inhibiting miR-139-5p

**DOI:** 10.1038/s41389-017-0008-4

**Published:** 2017-11-28

**Authors:** Zehua Bian, Jiwei Zhang, Min Li, Yuyang Feng, Surui Yao, Mingxun Song, Xiaowei Qi, Bojian Fei, Yuan Yin, Dong Hua, Zhaohui Huang

**Affiliations:** 1 Wuxi Cancer Institute, Affiliated Hospital of Jiangnan University, Wuxi, Jiangsu 214062 China; 2 Department of Pathology, Affiliated Hospital of Jiangnan University, Wuxi, Jiangsu 214062 China; 3 Department of Surgical Oncology, Affiliated Hospital of Jiangnan University, Wuxi, Jiangsu 214062 China; 4 Department of Medical Oncology, Affiliated Hospital of Jiangnan University, Wuxi, Jiangsu 214062 China

## Abstract

Long intergenic non-coding RNA 152 (*LINC00152*) is a recently identified tumor-promoting long non-coding RNA. However, the biological functions of *LINC00152* in colorectal cancer (CRC) remain unclear and require further research. The aim of the present study is to explore the roles of *LINC00152* in cellular function and its possible molecular mechanism. In this study, we discovered that *LINC00152* was overexpressed in CRC tissues and negatively related to the survival time of CRC patients. Functional analyses revealed that *LINC00152* could promote cell proliferation. Furthermore, *LINC00152* could increase the resistance of CRC cells to 5-fluorouracil (5-FU) by suppressing apoptosis. We also discovered that *LINC00152* could enhance cell migration and invasion. Mechanistic studies demonstrated that *LINC00152* could regulate the expression of NOTCH1 through sponging miR-139-5p and inhibiting its activity from promoting CRC progression and development. Altogether, our work points out a novel *LINC00152*/miR-139-5p/NOTCH1 regulatory axis in CRC progression and development.

## Introduction

Colorectal cancer (CRC) is the third most common cancer worldwide^1^. The occurrence and development of CRC involve a series of complex changes at the genetic and epigenetic levels^2^. Increasing number of studies have demonstrated that long non-coding RNAs (lncRNAs) are involved in the occurrence and development of CRC^3^.

LncRNAs are a kind of RNA molecules with more than 200 nucleotides and no protein translation ability. Recent advances have revealed the vital roles of lncRNAs in regulating tumorigenesis, and progression. Long intergenic non-coding RNA 152 (*LINC00152*) locates on chromosome 2p11.2 with 828 nt transcription length. *LINC00152* was overexpressed in tumor tissues and plasma of gastric cancer (GC) patients, and could promote GC cell proliferation and cell cycle progression through regulating EGFR and EZH2^4–7^. *LINC00152* also plays an oncogenic role in liver^8^, gallbladder^9^, and lung cancer^10^. In addition, *LINC00152* is likely to be an indicator of stress in a variety of cells^11^. These studies exhibit the key oncogenic role and complicated mechanisms of *LINC00152* in cancers. However, the detailed functions and mechanisms of *LINC00152* in CRC are mainly unclear.

In this study, we showed that *LINC00152* was upregulated in CRC, and correlated with poor survival. Functional analyses showed that *LINC00152* could enhance CRC growth, metastasis, and chemoresistance. Mechanistic studies demonstrated that *LINC00152* promotes tumorigenesis and progression via working as a competitive endogenous RNA (ceRNA) of miR-139-5p, which is a key tumor suppressive microRNA (miRNA)^12–18^. The present work reveals a novel regulatory pathway of *LINC00152*/miR-139-5p/NOTCH1 in CRC, suggesting that *LINC00152* is a new prognostic factor and potential therapeutic target in CRC.

## Results

### Overexpression of *LINC00152* in CRC associates with poor prognosis

**Fig. 1:**
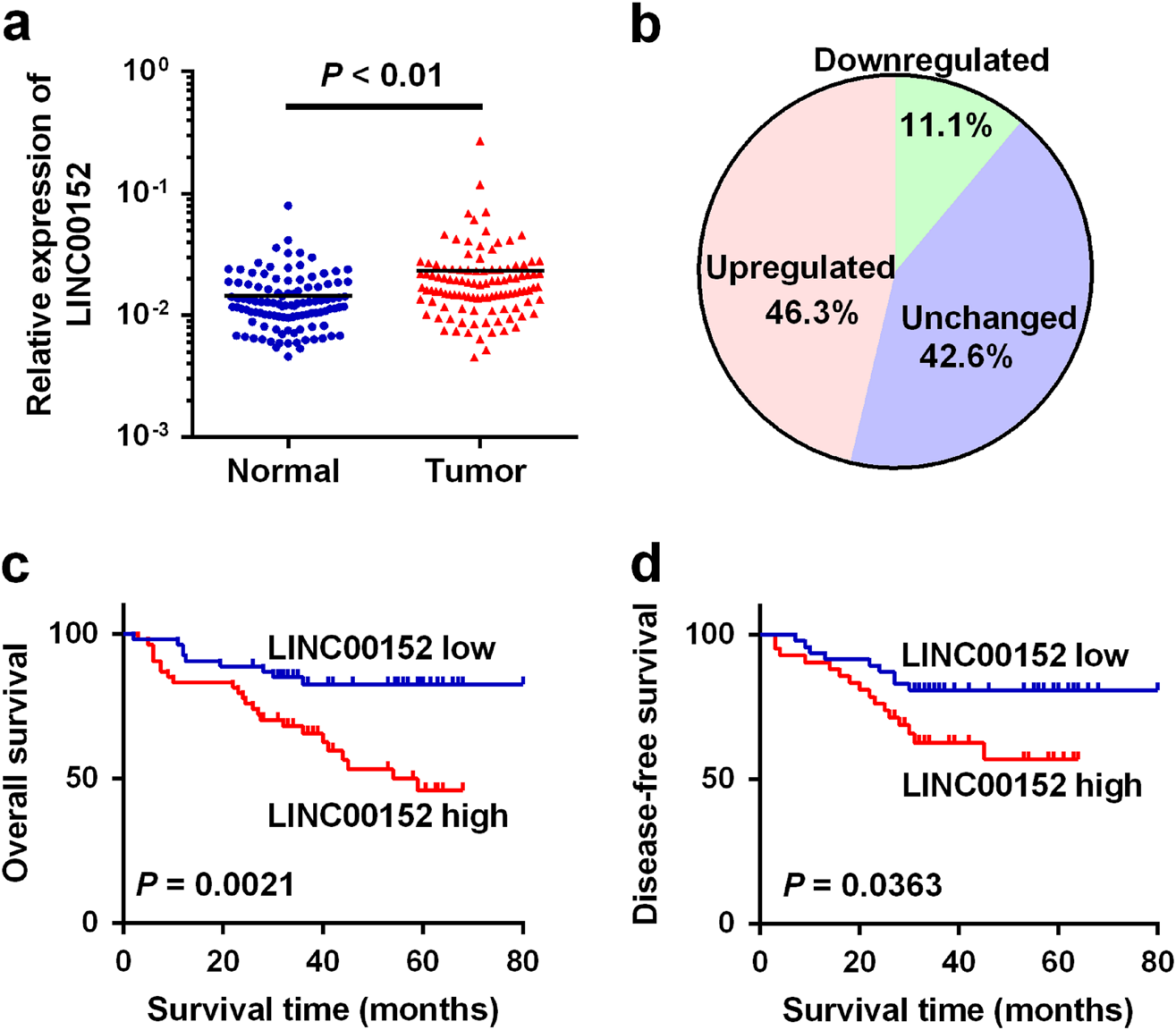
*LINC00152* is upregulated in tumor tissues of CRC **a** Relative expression levels of *LINC00152* in 108 paired CRC and NCTs were quantified by qRT-PCR. **b** *LINC00152* was upregulated (> 2-fold) in 46.3% of the CRC tissues compared with the NCTs. **c**, **d** Kaplan–Meier survival analysis of the overall survival and disease-free survival in two groups defined by low and high expression of *LINC00152* in patients with CRC

To study the role of *LINC00152* in CRC, we first detected its expression in 108 paired CRC tissues and noncancerous tissues (NCTs). The results revealed that *LINC00152* was obviously upregulated in CRC (*P* < 0.001, Fig. 1a), and 46.3% (50 of 108) of the CRC tissues showed > 2-fold upregulation of *LINC00152* compared with their NCTs (Fig. 1b).

To assess the potential association of *LINC00152* with clinicopathological features, we first divided the 108 patients into *LINC00152*-high and -low groups. We found that the *LINC00152* levels in CRCs were significantly correlated with tumor stage (*P* = 0.013), whereas no obvious correlation between *LINC00152* expression and other clinicopathological parameters was observed (Table 1).

**Table 1:** Correlation of the expression of LINC00152 with clinicopathologic features

Characteristics	LINC00152		*P*-value
	Low	High	
Ages (years)			
< 60	30	30	1.000
≥ 60	24	24	
Gender			
Male	31	33	0.434
Female	23	21	
Tumor size (cm)			
< 5	42	40	0.552
≥ 5	12	14	
Location			
Colon	30	29	0.847
Rectum	24	25	
Differentiation			
Well and moderately	48	44	0.283
Poorly	6	10	
Depth of tumor			
T1 + T2	13	6	0.083
T3 + T4	41	48	
Distant metastasis			
Absent	47	48	0.768
Present	7	6	
Tumor stage			
I + II	31	16	0.013
III + IV	23	38	

The survival analysis showed that patients in the *LINC00152*-high group showed a shorter survival time than those in the *LINC00152*-low group (46.614 ± 3.366 vs. 69.338 ± 3.271 months; log rank = 9.456, *P* = 0.0021, Fig. 1c). In addition, high *LINC00152* expression was also associated with poor disease-free survival (log rank = 4.383, *P* = 0.0363, Fig. 1d). Furthermore, multivariate analysis further identified that *LINC00152* was an independent prognosis factor for CRC (hazard ratio (HR) = 2.514, 95% confidence interval (CI) = 1.125-5.621, *P* = 0.025, Table 2).

**Table 2:** Univariate and multivariate regression analyses of parameters associated with prognosis of CRC patients

Characteristics	Subset	Univariate analysis		Multivariate analysis	
		*P*-value	HR (95% CI)	*P*-value	HR (95% CI)
Ages (years)	< 60/≥ 60	0.421	0.747 (0.367–1.519)	—	—
Gender	Male/female	0.818	0.921 (0.458–1.853)	—	—
Tumor size	< 5 cm/ ≥ 5 cm	0.385	1.355 (0.683–2.691)	—	—
Location	Colon/rectum	0.965	1.016 (0.512–2.016)	—	—
Differentiation	Well + moderately/poorly	0.019	2.499 (1.160–5.384)	0.061	2.103 (0.967–4.573)
Depth of tumor	T1 + T2/T3 + T4	0.035	8.471 (1.157–62.029)	0.169	4.083 (0.549–30.370)
Distant metastasis	Present/absent	0.404	0.602 (0.182–1.986)	—	—
Tumor stage	I + II/III + IV	0.000	10.017 (3.054–32.862)	0.001	7.140 (2.155–23.656)
LINC00152	High/low	0.001	3.825 (1.723–8.493)	0.025	2.514 (1.125–5.621)

### *LINC00152* promotes CRC cell proliferation

**Fig. 2:**
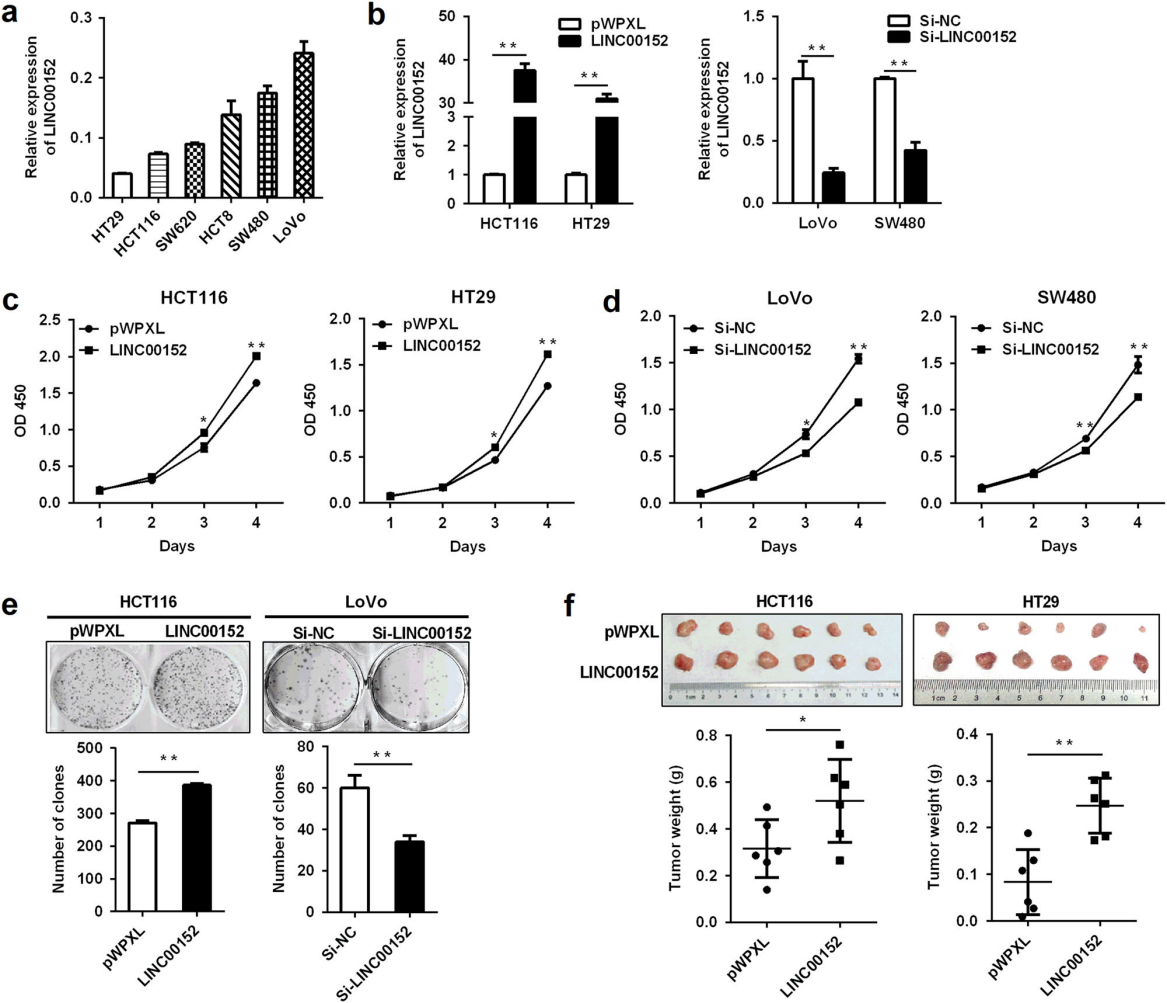
*LINC00152* promotes CRC cell proliferation *in vitro* and *in vivo* **a** Relative expression of *LINC00152* in CRC cell lines. **b** Validation of overexpression and knockdown efficacy of *LINC00152* in CRC cell lines by qRT-PCR. **c**, **d** Effects of *LINC00152* overexpression and downregulation on CRC cell proliferation were measured by a CCK-8 assay. **e** Effects of *LINC00152* overexpression and knockdown on colony formation in CRC cells. **f** *LINC00152* overexpression promoted CRC tumorigenesis in a xenograft mouse model. *P < 0.05; **P < 0.01

The expression analyses of *LINC00152* in six CRC cell lines showed that LoVo and SW480 have relatively high expressions of *LINC00152*, whereas HCT116 and HT29 have relatively low expressions of *LINC00152* (Fig. 2a). To investigate the biological functions of *LINC00152* in CRC, we overexpressed *LINC00152* in HCT116 and HT29 cells, and inhibited *LINC00152* expression in LoVo and SW480 cells (Fig. 2b). We observed that *LINC00152* overexpression significantly promoted CRC cell proliferation and colony formation. In contrast, decreased cell growth, and colony formation abilities were showed in *LINC00152*-silenced cells (Fig. 2c–e). Furthermore, ectopic *LINC00152* expression promoted CRC tumor growth *in vivo* (Fig. 2f). All these data reveal the growth-stimulating functions of *LINC00152* in CRC.

### *LINC00152* promotes cell cycle progression and confers resistance to 5-FU-induced apoptosis

**Fig. 3:**
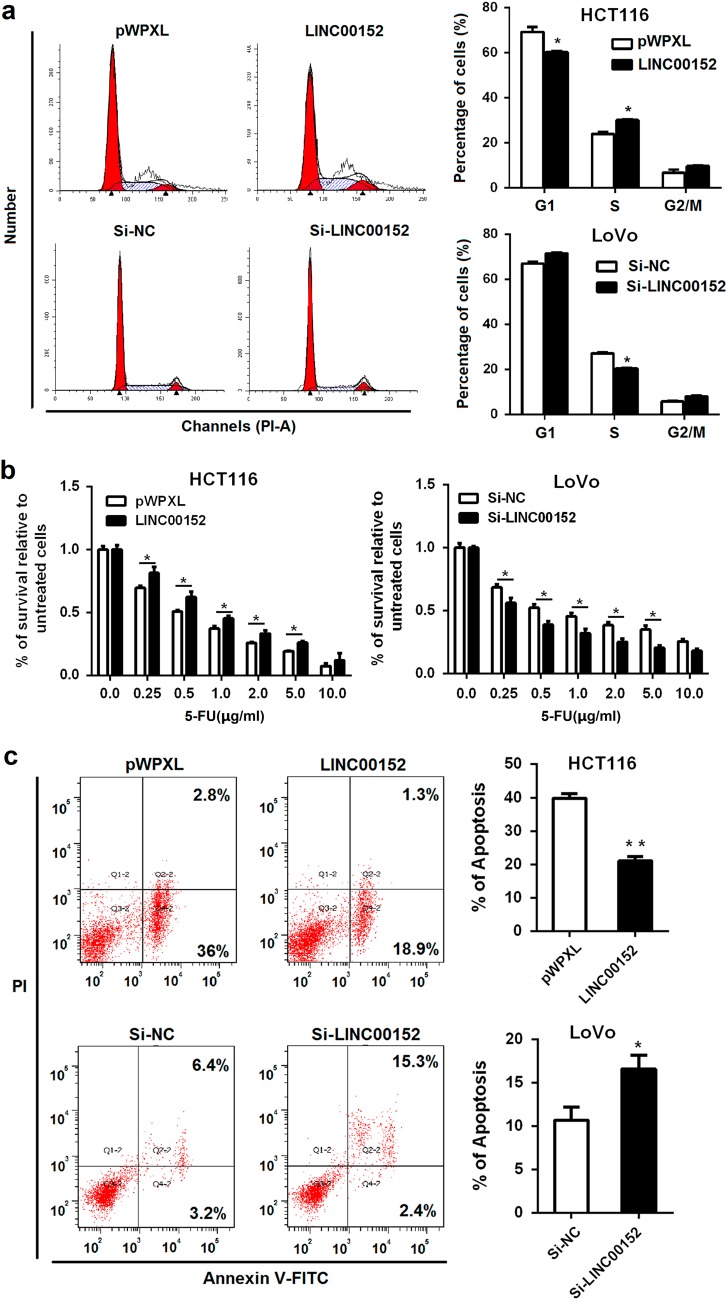
*LINC00152* promotes cell cycle progression and confers resistance to 5-FU-induced apoptosis **a** Cell cycle analyses were performed in HCT116 cells transfected with pWPXL-*LINC00152* and pWPXL, or LoVo cells transfected with si-*LINC00152* and si-NC. **b** *LINC00152* decreased the sensitivity of CRC cells to 5-FU. The IC50 of LINC00152-overexpressed HCT116 cells was significantly higher than that of the control (0.836 vs. 0.279 μg/ml), and the IC50 of LINC00152-silenced LoVo cells was lower than that the control (0.576 vs. 0.960 μg/ml). **c** Cell apoptosis analyses were performed in cell lines with *LINC00152* overexpression or knockdown. *P < 0.05; **P < 0.01

To investigate the mechanism mediating the growth-promoting functions of *LINC00152* in CRC, we measured the cell cycle distribution in the *LINC00152*-overexpressed and silenced CRC cells. As shown in Fig. 3a, ectopic *LINC00152* expression resulted in an increased number of cells in S phase, whereas *LINC00152* knockdown caused a decreased cell number in S phase, indicating the promotion of the cell cycle by *LINC00152*.

5-fluorouracil (5-FU) is a basic drug for CRC treatment, and we evaluated the effect of *LINC00152* on 5-FU sensitivity in CRC cells. After overexpression or knockdown of *LINC00152*, CRC cells were then assayed for their sensitivity to 5-FU by a CCK-8 assay. The results showed that ectopic *LINC00152* expression decreased the sensitivity of HCT116 cells to 5-FU, whereas *LINC00152* silencing increased the sensitivity to 5-FU in LoVo cells (Fig. 3b). Given the key role of apoptosis in cancer chemotherapy, we further measured the effect of *LINC00152* on 5-FU-induced apoptosis. The results showed that the *LINC00152* overexpression significantly antagonize 5-FU-induced apoptosis, whereas the *LINC00152* knockdown could augment apoptosis caused by 5-FU (Fig. 3c).

### *LINC00152* promotes CRC cell migration and invasion

**Fig. 4:**
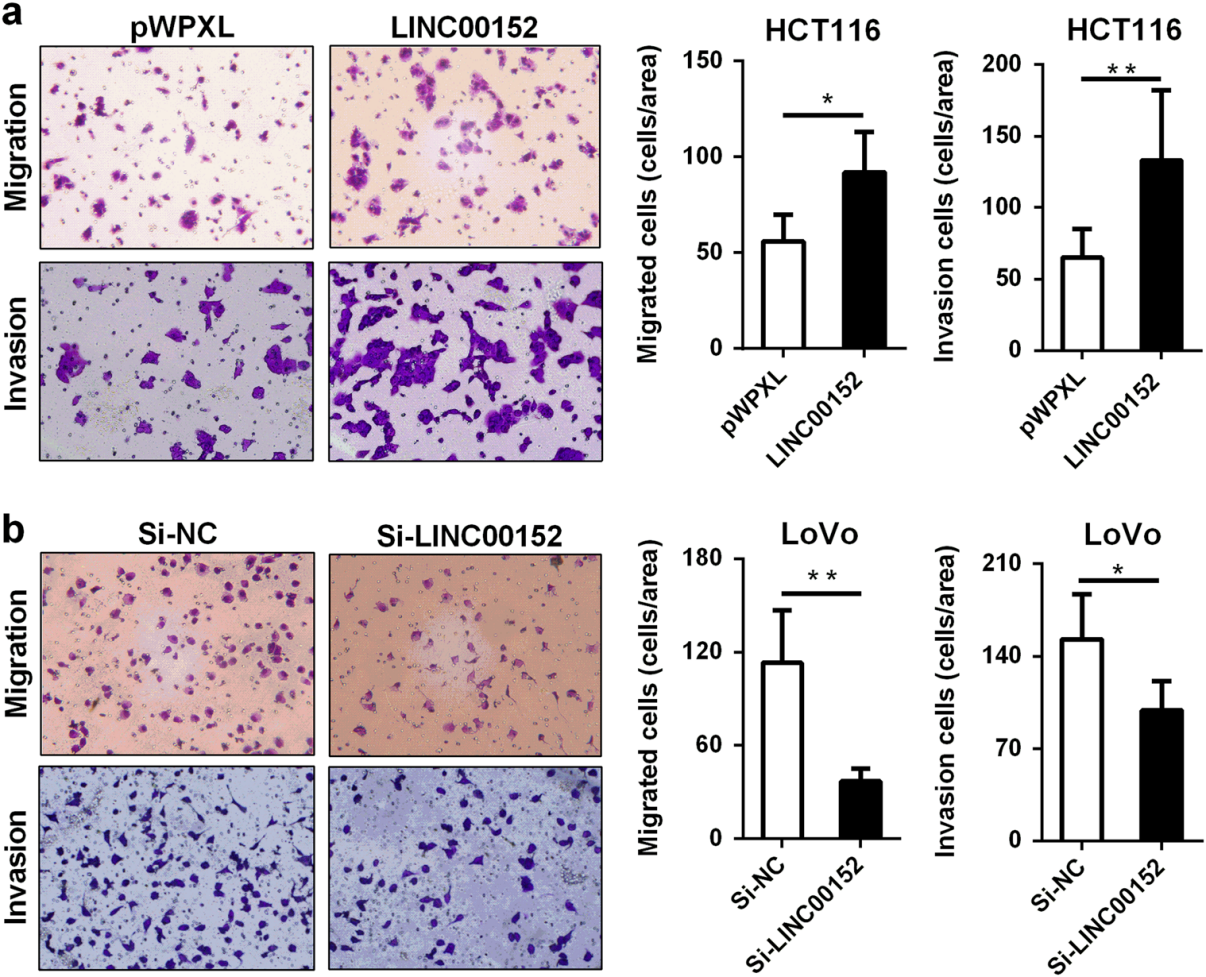
*LINC00152* promotes cell migration and invasion in CRC cells **a**, **b** Representative images and bargraphs depicting the migration and invasion ability of CRC cells with overexpressed or silenced *LINC00152*. *P < 0.05; **P < 0.01

Transwell assays were then performed to measure the impact of *LINC00152* on CRC metastasis. We observed that ectopic *LINC00152* expression significantly facilitated migration and invasion in HCT116 cells (Fig. 4a), whereas the *LINC00152* knockdown suppressed migration and invasion in LoVo cells (Fig. 4b).

### *LINC00152* sponges miR-139-5p

**Fig. 5:**
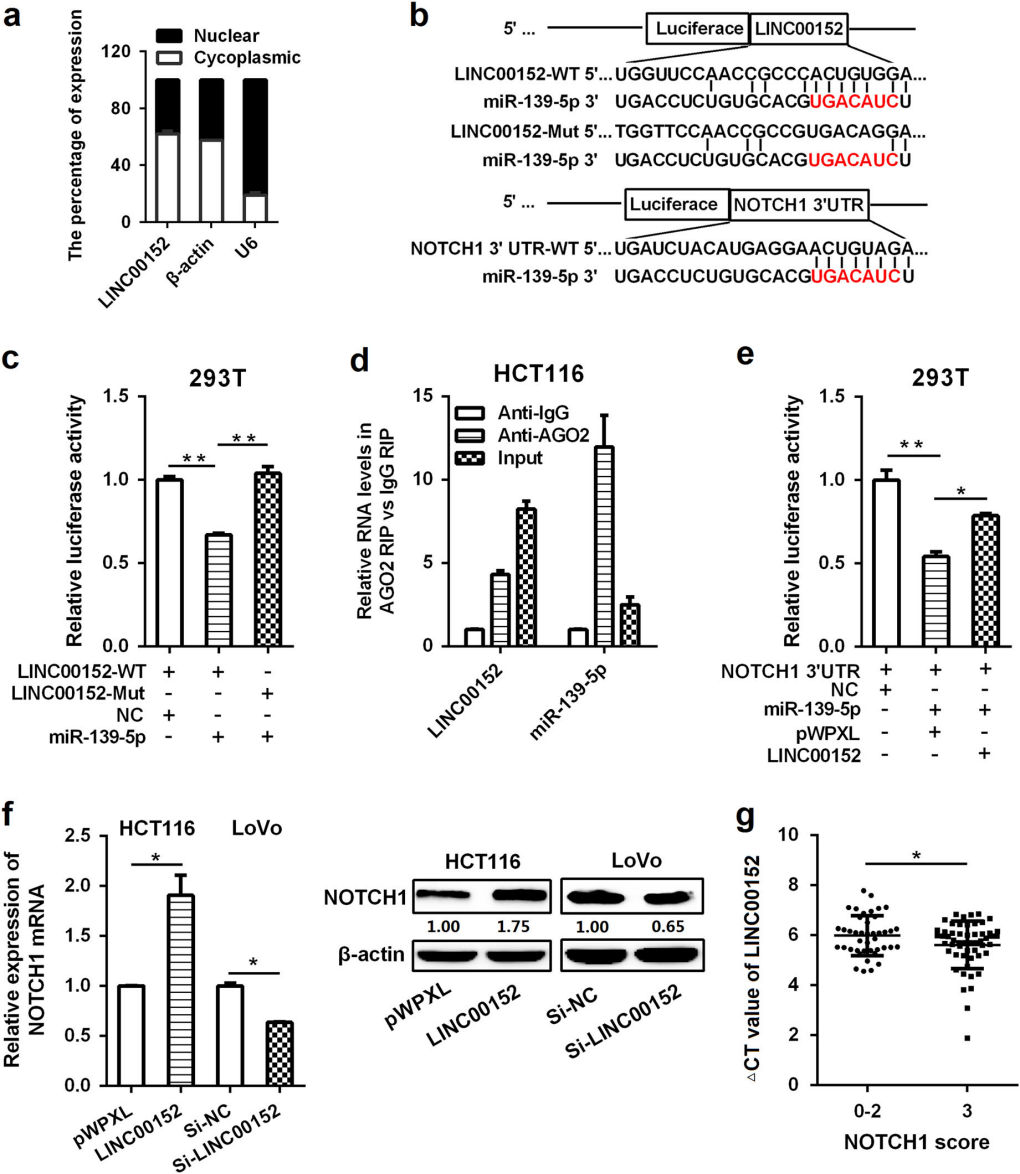
*LINC00152* sponges miR-139-5p and modulates NOTCH1 expression **a** Subcellular localization of *LINC00152* was determined by qRT-PCR in HCT116 cell line. **b** miR-139-5p-binding sequence in *LINC00152* and *NOTCH1* 3'UTR. A mutation was generated in *LINC00152* in the complementary site for miR-139-5p binding. **c** Luciferase activity of a luciferase reporter plasmid (pLuc) containing wild-type or mutant *LINC00152* co-transfected with miR-139-5p was determined using the dual luciferase assay. **d** Cellular lysates from HCT116 cells were used for RIP with an anti-Ago2 antibody or IgG antibody. The levels of *LINC00152* and miR-139-5p were detected by qRT-PCR. **e** MiR-139-5p and pLuc plasmid containing *NOTCH1* 3'UTRs were co-transfected with pWPXL-*LINC00152* or empty vector into 293T cells to verify whether *LINC00152* can function as a ceRNA of miR-139-5p. **f** The expression levels of NOTCH1 in HCT116 cells transfected with pWPXL-*LINC00152* and LoVo cells transfected with si-*LINC00152* were analyzed by qRT-PCR and western blot. **g** Correlation analysis between NOTCH1 and *LINC00152* expression. *P < 0.05; **P < 0.01

To investigate underlying mechanisms of *LINC00152* in CRC, we first measured the subcellular localization of *LINC00152* in HCT116 cells, and revealed that *LINC00152* was localized predominantly in the cell cytoplasm (Fig. 5a), suggesting that *LINC00152* may regulate tumorigenesis at the post-transcriptional level. LncRNAs could act as molecular sponges to modulate mRNAs expression by competitively binding their common miRNA responsive elements (MREs). Previous studies have proved that *LINC00152* could function as a ceRNA in human cancers^19–21^. We hypothesized that *LINC00152* could promote CRC tumorigenesis and progression by suppressing the functions of certain miRNAs. Based on the bioinformatics analysis and Xia’s work^22^, we found that *LINC00152* harbors a recognition sequence of miR-139-5p (Fig. 5b). In view of the opposite functions of miR-139-5p and *LINC00152* in CRC^12–18^, we intended to explore the potential relationship between them in CRC.

We first constructed reporter vectors containing *LINC00152* (pLuc-LINC00152-WT) or its mutant with mutations in the seed sequence of miR-139-5p (pLuc-LINC00152-Mut), and then evaluated this underlying correlation of miR-139-5p with *LINC00152* using luciferase reporter assays. We observed that miR-139-5p overexpression led to a marked inhibition in the reporter activity of pLuc-LINC00152-WT compared with that of pLuc-LINC00152-Mut (Fig. 5c), suggesting sequence-specific binding and inhibition of *LINC00152* by miR-139-5p. To further validate the potential binding of *LINC00152* to miR-139-5p, an RNA Immunoprecipitation (RIP) assay using an anti-Ago2 antibody was performed. The data exhibited that both *LINC00152* and miR-139-5p were obviously enriched in Ago2 complex, demonstrating that LINC00152 is included in miRNPs, probably through binding with miR-139-5p (Fig. 5d).

### *LINC00152* modulates NOTCH1 expression by competitively binding miR-139-5p

Previous studies have shown that miR-139-5p inhibit CRC tumorigenesis, development, and chemoresistance by regulating NOTCH1^12–15^. To ascertain whether the above-observed effects depend on the regulation of *LINC00152* on the miR-139-5p/NOTCH1 pathway, we first evaluated the relationship among *LINC00152*, miR-139-5p and NOTCH1 using luciferase assays. As a result, the overexpression of *LINC00152*, but not the vector control, blocked the inhibitory effect of miR-139-5p on the relative luciferase expression of pLuc-NOTCH1-3′UTR (Fig. 5e). These results confirmed that *LINC00152* abolishes the miR-139-5p-mediated repressive activity on NOTCH1 by competitively binding miR-139-5p. In addition, *LINC00152* knockdown significantly reduced the endogenous NOTCH1 expression in CRC cells (Fig. 5f). In contrast, NOTCH1 expression was obviously increased in *LINC00152* overexpressing CRC cells (Fig. 5f). A positive relationship was also observed between the levels of NOTCH1 and *LINC00152* in CRC tissues (Fig. 5g). These data demonstrate that *LINC00152* can regulate NOTCH1 activity by sponging miR-139-5p both in CRC cell lines and clinical CRC tumors.

### *LINC00152* exerts tumor-promoting function in CRC by regulating the miR-139-5p/NOTCH1 axis

**Fig. 6:**
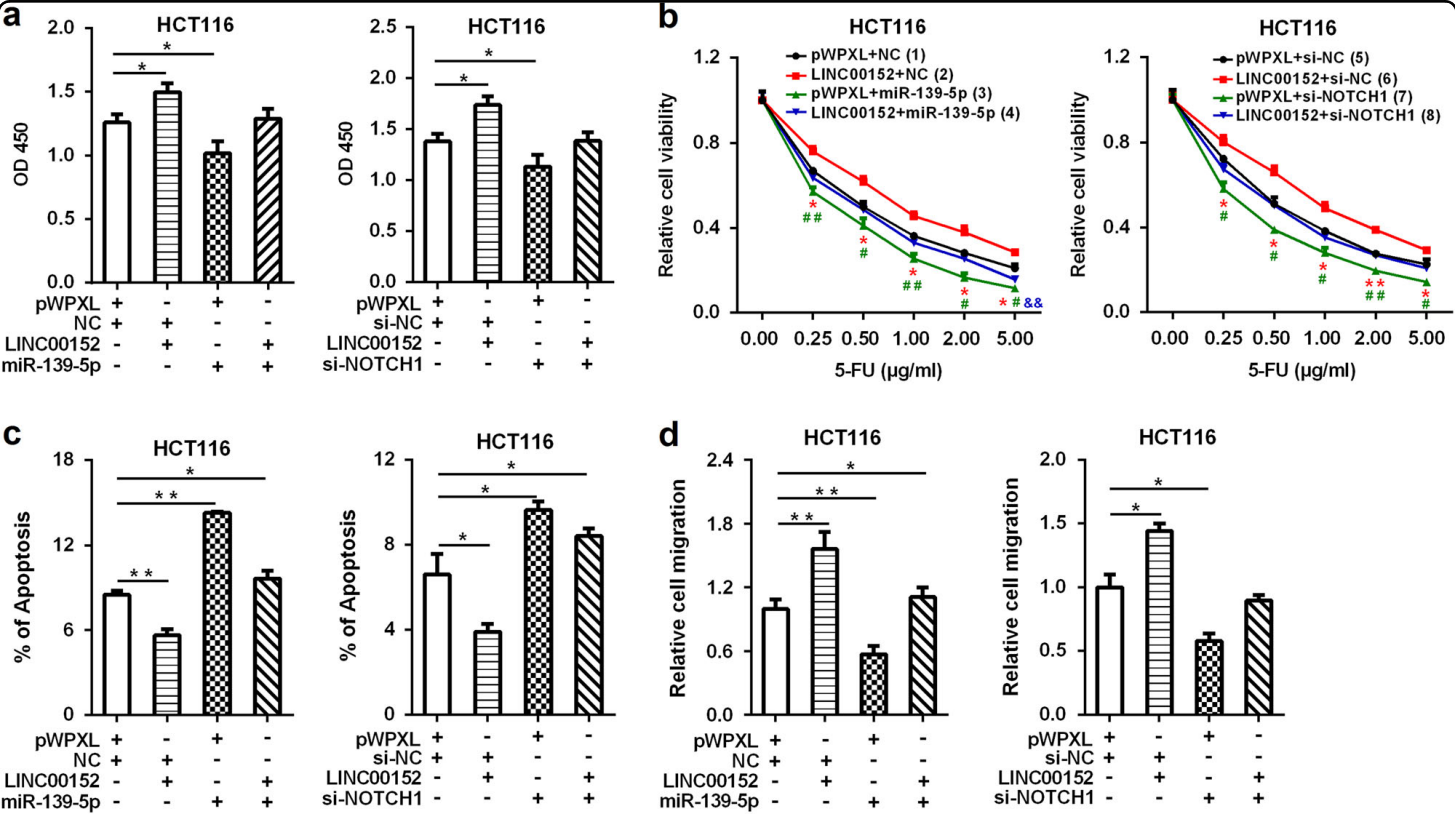
*LINC00152* exerts tumor-promoting function in CRC by regulating the miR-139-5p/NOTCH1 axis **a** The increased cell viability in pWPXL-*LINC00152* transfected CRC cells was abolished by ectopic miR-139-5p expression or *NOTCH1* knockdown. The cell viability was measured by a CCK-8 assay. **b** Increased 5-FU resistance in pWPXL-*LINC00152* transfected CRC cells was abolished by ectopic miR-139-5p expression or *NOTCH1* knockdown. The IC50s for group (1) to (8) were 0.632, 1.180, 0.327, 0.512, 0.564, 1.014, 0.329, and 0.466 μg/ml, respectively. **c** Overexpression of *LINC00152* decreased 5-FU-induced apoptosis, which was partly blocked by ectopic miR-139-5p expression or *NOTCH1* knockdown. **d** Ectopic miR-139-5p expression or *NOTCH1* knockdown could partly block *LINC00152*-induced cell migration. * or ^#^*P < *0.05; **or ^##^or ^&&^*P* < 0.01 (* or **: (1) vs. (2) or (5) vs. (6); ^#^ or ^##^: (1) vs. (3) or (5) vs. (7); ^&&^: (1) vs. (4))

Both miR-139-5p and NOTCH1 could regulate cell growth, apoptosis, and invasion in CRC^12–18, 22^. To investigate whether *LINC00152* exerts tumor-promoting functions in CRC by modulating the miR-139-5p/NOTCH1 axis, we first checked the effects of miR-139-5p and NOTCH1 on *LINC00152*-induced cell proliferation, and observed that miR-139-5p overexpression or *NOTCH1* knockdown blocked the *LINC00152*-induced CRC cell growth (Fig. 6a). We then evaluated the effects of miR-139-5p/NOTCH1 signaling on the *LINC00152*-induced 5-FU resistance in CRC cells. As shown in Fig. 6b, ectopic miR-139-5p expression or *NOTCH1* knockdown significantly reversed the *LINC00152*-induced 5-FU resistance and counteracted the apoptosis-inhibiting effects of *LINC00152* in CRC cells (Fig. 6c). In addition, the increased cell mobility in *LINC00152* overexpressing CRC cells was also reversed by miR-139-5p overexpression or *NOTCH1* knockdown (Fig. 6d). Altogether, these data demonstrate that *LINC00152* exerts tumor-promoting functions in CRC, at least partly, through sponging miR-139-5p and then regulating NOTCH1.

## Discussion

In this study, we observed that *LINC00152* expression is obviously increased in clinical CRC tissues, and is correlated with tumor stage and poor patient survival. Functionally, we revealed that *LINC00152* promotes CRC growth, metastasis, and induces 5-FU resistance. Moreover, we further demonstrated that *LINC00152* executes tumor-promoting functions by sponging miR-139-5p and then modulating NOTCH1 in CRC.

Numerous studies have revealed varied regulatory roles of lncRNAs in human diseases, especially in tumorigenesis and development^23^. For example, our previous work revealed that *UCA1* could promote cell proliferation and 5-FU chemoresistance in CRC via competitively inhibiting miR-204-5p^24^. *LINC00152* is recently identified cancer-related lncRNA that play oncogenic roles in several kinds of human cancers, especially in digestive tract tumors^4–9, 11^. Yue et al.^19^ reported that *LINC00152* expression is increased in CRC. Interestingly, in contradictory to their conclusions, a recently published work demonstrated that *LINC00152* is downregulated in CRC, inhibits viability and promotes apoptosis of CRC cells^25^. Here, we demonstrated that *LINC00152* expression was obviously increased in CRC and correlated with patient’s survival, which was also observed by Yue et al.^19^. Our detailed functional studies revealed the promoting effects of *LINC00152* on CRC growth and metastasis, which is coincident with the oncogenic role of *LINC00152* in GC^4–7^, liver cancer^8^, gallbladder cancer^9, 26^, and clear cell renal cell carcinoma^27^. In addition, we also showed that *LINC00152* confers resistance to 5-FU-induced apoptosis, which was similar to that reported by Yue et al.^19^. In their study, Yue et al. demonstrated that *LINC00152* works as a ceRNA of miR-193a-3p to induce oxaliplatin resistance. These data demonstrate that *LINC00152* is a key lncRNA with extensive tumor-promoting functions in human cancers.

Several studies have reported that *LINC00152* promotes tumor development and progression by regulating several key tumor-related pathways, including EGFR, mTOR, and PI3K/AKT signaling^4, 8, 9^. Recent studies revealed a new mechanism of lncRNA by acting as ceRNA^20^. In this situation, lncRNAs can block the repression of miRNA on its target genes by competitively binding their common MREs^28^. *LINC00152* could bind several miRNAs in cancer cells, including miR-138, miR-376c-3p, and miR-193a-3p^19, 25, 26^, suggesting that ceRNA is a key mechanism by which *LINC00152* regulates tumorigenesis and development.

Due to the upregulation and tumor-promoting role of *LINC00152* in CRC, it is reasonably concluded that *LINC00152* promotes CRC development and progression by inhibiting tumor suppressive miRNAs. Based on previous works by us and other researchers, miR-139-5p levels are markedly reduced in CRC, and has exact opposite functions to those of *LINC00152*^12–18^. MiR-139-5p can repress CRC growth, metastasis, and chemoresistance by regulating several genes, such as NOTCH1, BCL2, and AMFR^12–18^. MiR-139-5p was reported to play a suppressive role in other cancers, including gastric, breast, and hepatocellular carcinoma^29^. As a key member of the NOTCH family, NOTCH1 is frequently upregulated in human cancers, including CRC^30^. Previous researches have proved that miR-139-5p can regulate CRC growth, metastasis, stemness, and chemoresistance via targeting NOTCH1^12–15, 31^. We speculated that *LINC00152* exerts its functions by regulating the miR-139-5p/NOTCH1 pathway. As expected, both the luciferase and RIP assays confirmed the binding of *LINC00152* to miR-139-5p. Subsequent functional and mechanistic assays proved that *LINC00152* regulates CRC development, progression, and drug resistance by competitively sponging miR-139-5p and then restoring NOTCH1 activity.

In summary, our work shows that *LINC00152* is upregulated in CRC, correlated with patients’ survival and appears to be a potential biomarker for predicting chemoresistance. *LINC00152* contributes to the tumorigenesis, progression, and chemoresistance of CRC by inhibiting miR-139-5p, uncovering a novel ceRNA network of *LINC00152*/miR-139-5p/NOTCH1 in CRC cells. These data suggest that targeting *LINC00152* may be a promising therapeutic strategy for CRC.

## Materials and methods

### Clinical samples

A total of 108 paired human CRC tissues and NCTs were collected with informed consent at Affiliated Hospital of Jiangnan University, and the detailed patient information are shown in Table 1. This study was carried out under the permission of the Clinical Research Ethics Committees of Affiliated Hospital of Jiangnan University.

### Cell lines

HEK-293T and six CRC cell lines (HCT8, HT29, LoVo, HCT116, SW480, and SW620) were obtained from the American Type Culture Collection. These cells were maintained in Dulbecco's modified Eagle's medium supplemented with 10% fetal bovine serum (Gibco, USA) and have been recently authenticated.

### RNA isolation and quantitative reverse transcription (RT)-PCR assays

Total RNA was isolated with RNAiso Plus (Takara, Japan). Cytoplasmic and nuclear RNA was purified using PARIS^TM^ Kit (Ambion, USA). Complimentary DNA was synthesized using the HiFiScript 1st Strand cDNA Synthesis Kit (CWBIO, China). Real time RT-PCR was performed using an UltraSYBR Mixture (CWBIO).

### Vector construction and siRNA

*LINC00152* was synthesized at GENEray Biotechnology (China) and was inserted into the lentivirus vector pWPXL. The fragment of *LINC00152* with miR-139-5p-binding site and the *NOTCH1* 3'UTR were cloned into pLuc. The *LINC00152* with the mutated seed sequence of miR-139-5p was constructed by an overlap extension PCR^32^. The primers used in vector construction are shown in Supplementary Table 1. The siRNAs of *LINC00152* and *NOTCH1* were purchased from GenePharma (China).

### Generation of cell lines with stable overexpression of *LINC00152*

HEK-293T cells were transfected with pWPXL-LINC00152 (or pWPXL), pMD2G, and ps-PAX2 plasmids using Lipofectamine 2000 (Invitrogen, USA). These virus particles were centrifuged and filtered to infect HCT116 and HT29 cells to generate corresponding stable cells.

### Cell proliferation and colony formation assays

Cell Counting Kit 8 (CCK-8, Beyotime, China) was used to measure cell viability. A colony formation assay was performed as we previously described^33^.

### Cell cycle and apoptosis analyses

The cell cycle and apoptosis analyses of *LINC00152*-overexpressed and silenced CRC cells were applied using the Cell Cycle and Apoptosis Detection Kit purchased from CWBIO.

### Cell migration and invasion assay

Transwell assays were performed to measure cell migration and invasion using Boyden chambers (8-mm pore size, BD Biosciences) as we previously described^33^.

### Xenograft tumor assay

Twenty-four male athymic nude BALB/c mice at 5 weeks of age were randomly divided into four groups, and the number of mice is determined according to prior experience of our laboratory. HCT116 cells stably expressing *LINC00152* or the bank vector were subcutaneously injected into flank of nude mouse. Four (HCT116) or six weeks (HT29) after injection, these mice were sacrificed to measure the growth of subcutaneous tumors. The investigator was blinded to group allocation during the experiments. All animal experiments were approved by the Clinical Research Ethics Committees of our Hospital.

### Luciferase reporter assay

HEK-293T cells were co-transfected with pLuc, pRL-CMV, miR-139-5p mimics (negative control, NC), and pWPXL-LINC00152 (pWPXL). These cells were then assayed for luciferase activity using the Dual-Luciferase® Reporter Assay System (Beyotime, China).

### RNA Immunoprecipitation (RIP) assay

A RIP assay was performed using the EZ-Magna RIP Kit (Millipore, USA) as we previously described^24^.

### Western blotting

Total protein was separated by 8% (or 10%) sodium dodecyl sulfate polyacrylamide gel electrophoresis and transferred to a PVDF membrane. After blocking with non-fat milk, the polyvinylidene difluoride membrane was incubated with a rabbit anti-human NOTCH1 antibody (1:1000, 20687-1-AP, Proteintech, USA) or a mouse anti-β-actin antibody (1:1000, AA128, Beyotime, China).

### Statistical analyses

Data were presented as the mean ± s.d. Student’s *t*-test, the Mann–Whitney *U*-test and the *χ*2 test were performed to analyze the differences among different groups. The differences in survival rates were determined by the Kaplan–Meier method and compared by the log-rank test. HRs and 95% CIs were calculated by a Cox proportional hazards model. *P*-values < 0.05 were considered statistically significant.

## Electronic supplementary material

Supplementary Table S1. Primer sequences
