# Correction: Long non-coding RNA LINC00152 promotes cell proliferation, metastasis, and confers 5-FU resistance in colorectal cancer by inhibiting miR-139-5p

**DOI:** 10.1038/s41389-018-0067-1

**Published:** 2018-08-16

**Authors:** Zehua Bian, Jiwei Zhang, Min Li, Yuyang Feng, Surui Yao, Mingxun Song, Xiaowei Qi, Bojian Fei, Yuan Yin, Dong Hua, Zhaohui Huang

**Affiliations:** 1 Wuxi Cancer Institute, Affiliated Hospital of Jiangnan University, Wuxi, Jiangsu 214062 China; 2 Department of Pathology, Affiliated Hospital of Jiangnan University, Wuxi, Jiangsu 214062 China; 3 Department of Surgical Oncology, Affiliated Hospital of Jiangnan University, Wuxi, Jiangsu 214062 China; 4 Department of Medical Oncology, Affiliated Hospital of Jiangnan University, Wuxi, Jiangsu 214062 China

Correction to: *Oncogenesis*; 10.1038/s41389-017-0008-4; published online 28 November 2017

This article was originally published under Nature Research's License to Publish, but now has been made available under a CC BY 4.0 license. The PDF and HTML versions of the Article have been modified accordingly.

