# Supplementary material for: Long non-coding RNA LINC00152 promotes cell proliferation, metastasis, and confers 5-FU resistance in colorectal cancer by inhibiting miR-139-5p
Source: Oncogenesis. 2017 Nov 28;6(11):395. doi: 10.1038/s41389-017-0008-4 (PMC5868057; doi:10.1038/s41389-017-0008-4)
Supplement: Supplementary file 1 — Supplementary Table S1. Primer sequences [file 41389_2017_8_MOESM1_ESM.docx]

**Supplementary Table S1. Primer sequences**

| **Primers** | **Sequences** | **Products length** |
| --- | --- | --- |
| **Primers for real time PCR** | | |
| LINC00152-A | CGGGCAACAGGTAGAGGT |  |
| LINC00152-S | CTGGGAGATGAAACAGGAAG | 100 |
| NOTCH1-A | GCAGTCAGGCGTGTTGTTC |  |
| NOTCH1-S | GGCACTTTCTGTGAGGAGGA | 147 |
| β-actin-F | AGTGTGACGTGGACATCCGCAAAG |  |
| β-actin-R | ATCCACATCTGCTGGAAGGTGGAC | 220 |
| **Pimers for construction luciferase reporter vectors** | | |
| LINC00152-Y-F | CGGAATTCTCTGTGATGTCCCCAGTGAT | 418 |
| LINC00152-Y-R | CGGGATCGAAGGACAAGGGATTAAGACACA |  |
| LINC00152-M-R1 | CCTCAGAGTCCTGTCACGGCGGTTGGAACCAG |  |
| LINC00152-M-F2 | TTCCAACCGCCGTGACAGGACTCTGAGGCCTCTGC |  |
| F1 | GACGAAGTACCGAAAGGTCT |  |
| R2 | GCTGGCAACTAGAAGGCA |  |
| F3 | TCCTCATAAAGGCCAAGAA |  |
| R3 | CTAGCATTTAGGTGACACTATAGA |  |
